# Epithelial stratification shapes infection dynamics

**DOI:** 10.1371/journal.pcbi.1006646

**Published:** 2019-01-23

**Authors:** Carmen Lía Murall, Robert Jackson, Ingeborg Zehbe, Nathalie Boulle, Michel Segondy, Samuel Alizon

**Affiliations:** 1 Laboratoire MIVEGEC (UMR CNRS 5290, IRD, UM), Montpellier, France; 2 Probe Development and Biomarker Exploration, Thunder Bay Regional Health Research Institute, Thunder Bay, Ontario, Canada; 3 Biotechnology Program, Lakehead University, Thunder Bay, Ontario, Canada; 4 Department of Biology, Lakehead University, Thunder Bay, Ontario, Canada; 5 Pathogenesis and Control of Chronic Infections, INSERM, EFS, Université de Montpellier, Montpellier, France; Duke University, UNITED STATES

## Abstract

Infections of stratified epithelia contribute to a large group of common diseases, such as dermatological conditions and sexually transmitted diseases. To investigate how epithelial structure affects infection dynamics, we develop a general ecology-inspired model for stratified epithelia. Our model allows us to simulate infections, explore new hypotheses and estimate parameters that are difficult to measure with tissue cell cultures. We focus on two contrasting pathogens: *Chlamydia trachomatis* and Human papillomaviruses (HPV). Using cervicovaginal parameter estimates, we find that key infection symptoms can be explained by differential interactions with the layers, while clearance and pathogen burden appear to be bottom-up processes. Cell protective responses to infections (e.g. mucus trapping) generally lowered pathogen load but there were specific effects based on infection strategies. Our modeling approach opens new perspectives for 3D tissue culture experimental systems of infections and, more generally, for developing and testing hypotheses related to infections of stratified epithelia.

## Author summary

Many epithelia are stratified in layers of cells and their infection can result in many pathologies, from rashes to cancer. It is important to understand to what extent the epithelial structure determines infection dynamics and outcomes. To aid experimental and clinical studies, we develop a mathematical model that recreates epithelial and infection dynamics. By applying it to a virus, human papillomavirus (HPV), and a bacteria, chlamydia, we show that considering stratification improves our general understanding of disease patterns. For instance, the duration of infection can be driven by the rate at which the stem cells of the epithelium divide. Having a general model also allows us to investigate and compare hypotheses. This ecological framework can be modified to study specific pathogens or to estimate parameters from data generated in 3D skin cell culture experiments.

## Introduction

Stratified epithelia cover most of the human body’s exterior and line the inner cavities, such as the mouth and vagina. Localized (non-systemic) infections of these epithelia can cause a wide range of conditions that collectively represent a major burden on global public health systems. For instance, skin conditions are ranked 4th in global years lost due to disability (YLDs) and are in the top 10 most prevalent diseases globally [1]. Infections (viral, fungal, bacterial, etc.) are either the etiological agents or are secondary opportunistic infections (e.g. scabies, eczema) of many skin conditions and thus play a major role in their burden and outcomes. While stratified epithelia are often the first line of defense against infections [2], their cells are the primary target for many viruses or bacteria. This is why understanding epithelial life-cycles, signaling, and dynamics is an active line of research [3].

Epithelial infections are very heterogeneous in their outcomes, ranging from short sub-clinical acute infections to chronic pathologies [1]. Our hypothesis is that the stratified structure is one of the keys to understanding these patterns. Though experimental and clinical methods used for studying these infections are increasingly quantitative (e.g. flow cytometry or -omics technologies), theoretical frameworks for understanding infection properties and dynamics in stratified epithelia are lacking since most models consider infections of monolayers or blood. Here, we build on the analogy between a host and an ecological system [4, 5] to investigate how the stratification of the epithelium drives infection dynamics. We focus on keratinocyte epithelia as an example as it is a well-studied stratified system with important public health implications.

Localized infections of stratified epithelia such as the cervicovaginal mucosa are involved in a range of health concerns, such as decreasing fertility [6–9] or carcinogenesis [10]. Studying the cervical epithelium has greatly helped improve women’s health [11] and histological studies of cervical infections have characterized both healthy and diseased cells. The ectocervix is a non-keratinized stratified epithelium that acts as an important barrier to prevent infections from entering the upper part of the female genital tract and affecting fertility. The tight packing of the epithelial cells and their migration to the surface are believed to prevent bacteria and viruses from reaching the dermis [12]. Furthermore, the continual production of surface mucus is thought to aid in trapping and removing invaders [13]. Studying these processes using tractable experimental systems has been a challenge given the complexity of recreating stratified epithelia with realistic features, but this is changing rapidly [14]. Mathematical modeling can aid this experimental work by helping to estimate parameters such as changes in cell migration or mucus production rate.

The vast majority of mathematical models of within-host dynamics focus on virulent viruses causing systemic infections, such as HIV (for a review, see [15]), but some investigate pathogens that only (or mainly) target epithelia such as *Chlamydia* [16–20], HPV [21–24], Epstein-Barr Virus (EBV) [25, 26] or HSV [27]. A common feature of these models is that they focus on the pathogen and the associated immune response, while largely overlooking the epithelium itself. As a consequence, with few exceptions (e.g. [23]), they assume that the population of cells infected by the pathogen is homogeneous and not structured. We take an ecological approach to model the stratified epithelium to investigate the effect of the structure of the life cycle of the host cells on infection dynamics. The analogy between ecological systems and within-host interactions is not new (e.g. [4]), but it is becoming increasingly common and has underlaid successful quantitative tools for understanding viral kinetics [15, 28] and drug resistance [29].

From an ecological perspective, the stratified epithelial structure can be viewed as having stages or age structure (herein called ‘*stage-structure*’), meaning the full life-cycle of an individual cell is divided up into stages (or ages). Therefore, populations of one stage give rise to another in a successive fashion. Ecological populations with stage-structure have been shown to have rich dynamics [30]. If resource populations (species low in a food chain) are stage-structured, then the resulting dynamics can impact the entire ecological system [30–32]. Generally, oversimplifying (e.g. not considering the stage-structure of the resource) or not considering the resources is known to potentially lead to incorrect predictions about the behavior of the system [33]. Similar importance has been shown in host-pathogen systems. For instance, by combining mathematical models with experimental data Mideo *et al*. showed that differences between *Plasmodium chabaudi* strains could be most parsimoniously explained by their different affinity for erythrocytes of different ages, as well as differences in erythropoiesis, i.e. in how red blood cells are made [34]. Target cell heterogeneity has also been put forward to explain the HIV co-receptor switch [35]. While we pursue this analogy, we insist that stratified epithelia exhibit features that differ from traditional populations. For instance, differentiated keratinocytes (or ‘adults’) do not reproduce to make stem cells (or ‘juveniles’) the way free-living species do. Additionally, the epithelium self-regulates its dynamics as a means to maintain homeostasis, which involves the maintenance of constant numbers of cells by physiological processes, such as states of dormancy, proliferation and signaling [3]. Together, this calls for a system-specific approach.

Having a framework for epithelial dynamics allows us to simulate infections. For this, we chose two prevalent stratified epithelium infections with very different biological features: Human papillomaviruses (HPVs) and *Chlamydia trachomatis* bacteria. In the United States alone, more than 1.5 million cases of *C. trachomatis* were reported to the Center for Disease Control (CDC) in 2017 and HPVs are the most common sexually transmitted infection in the country [36]. Most HPVs are, in fact, not sexually transmitted and are part of a large family of viruses that infect stratified epithelia throughout the body (of the mucosal and or cutaneous tissues) and are considered part of our virome [37, 38]. While both *Chlamydia* and HPVs replicate intra-cellularly, these two infections exhibit contrasting strategies for infecting the squamous epithelium: HPVs cause non-lytic basal-up infections, whereas chlamydia infections are from the surface-down and are lytic. As mentioned, there are some previous mathematical models of both HPV and chlamydia [16–24] and, importantly, the biology of these two pathogens have been considerably studied, with well characterized life-histories (HPVs [37] and *Chlamydia* [39]). Consequently, this provided us with peer-reviewed parameter estimates, biologically grounded assumptions and previous results from mathematical models without epithelium stage-structure with which to compare our results. Finally, to maintain focus on the epithelium, we used a simple model for the immune response, as in earlier studies (e.g. on HSV, [27]).

We address to what extent epithelium dynamics affect infection dynamics and as a result determine infection outcomes. First, we introduce a general epithelium model, which we calibrate using existing data, as well as original cell culture data from a spontaneously immortalized human cell line (NIKS) [40]. With this data we infer parameters that are difficult to measure, such as the fraction of symmetric cell divisions. We then ‘infect’ this epithelial model with chlamydia, wart-associated HPVs and oncogenic (high-risk, HR) HPVs to investigate how protective measures by the epithelium affect infection load and duration, while identifying the parameters that control key infection traits. We find that epithelium stratification plays a key role in the dynamics and outcomes of these infections.

## Results

### Uninfected epithelial dynamics

**Fig 1:**
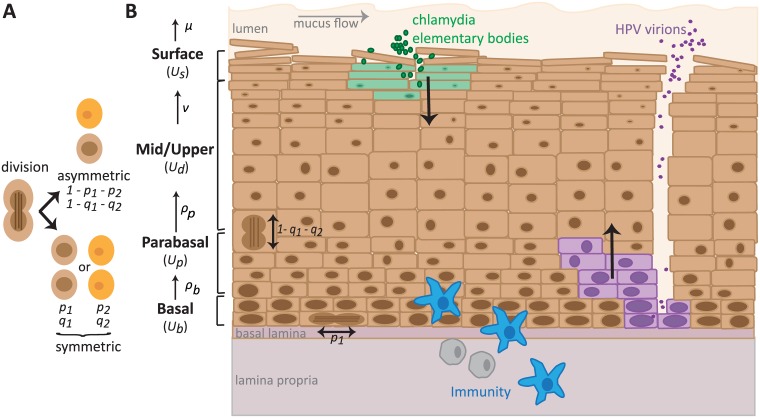
Dynamics in the stratified epithelium. A) Basal and parabasal cells can divide either asymmetrically (1 − *p*_1_ − *p*_2_ and 1 − *q*_1_ − *q*_2_ respectively) or symmetrically, which result in two daughter cells of either the same (*p*_1_ or *q*_1_) or different phenotype as the mother cell (*p*_2_ or *q*_2_). B) The squamous epithelium is abstracted into a basal, a parabasal, a mid-upper and a surface layer. Proliferation (*ρ*) and maturation (*ν*) rates determine the movement of cells up the layers. Cells die and are shed (*μ*). ***Chlamydia trachomatis*** (in green) infects the most superficial live cells underneath the mucus and surface dying cells. Once inside a cell, the elementary bodies (EB) change into reticulate bodies, which go through several rounds of replication, and then change back into EBs that are released upon cell death. ***Human papillomaviruses*** (in purple) must infect basal cells to establish an infection, thus usually requiring a microabrasion. The virus is non-lytic and replicates in host cells as they follow their natural life-cycle up the epithelium column. Progeny virions are released once the cell dies at the surface. Immune cells (in blue) enter the epithelium from the basal layer.

Our model abstracts the stratified epithelium into four phenotypically distinct populations, relevant to clinical and experimental models of the epithelium: stem-like cells in the basal and parabasal layers, and differentiated cells in the mid and surface layers (Fig 1 and equation system 1). These phenotypes can be identified experimentally using immunofluorescence techniques that target genes or proteins expressed differentially as the cells mature and move up the epithelial column. The model is sufficiently generic that it can represent any stratified squamous epithelium, keratinized or not. We considered the cervicovaginal mucosa as an example to parametrize and infect. The model includes 7 parameters, of which 4 are inferred from cervical autoradiographic experiments done in 1970 [41] and one, *N*_*b*_, is a scaling parameter describing the surface of the basal monolayer considered. The two remaining capture the difference in symmetric divisions probabilities by the basal and parabasal cells (Δ*p* is fixed at zero and Δ*q* is free and calibrated). All the parameters are listed in Table 1.

**Table 1:** Parameter descriptions for epithelial model, default values, biologically realistic ranges and estimated values. Literature estimates (a) are for cervicovaginal epithelia, while data-derived estimates (b) are for NIKS cell cultures, which are a common cell-line used to model these systems, but are not identical to *in vivo* cells in the cervicovaginal squamous epithelium (for example the latter cannot form keratinized layers). Thus, estimates are not expected to be identical. Additionally, (a) parameters are measured from systems already at homeostasis, while in (b) the cultures grow-up from a single layer. Parameter values that were chosen for the results to be biologically consistent are labelled as ‘calibrated’. The values ‘fixed*’ and ‘estimated*’ were derived using data (see S1 Text).

		Default	Range	Ref
	***a. in vivo estimates (literature)***			
*N*_*b*_	Total number of basal cells	10^3^	[10^2^; 10^5^]	fixed
*ρ*_*b*_	Basal cell replication rate (day^−1^)	0.03	[0.03; 0.07]	[41]
*ρ*_*p*_	Parabasal cell replication rate (day^−1^)	0.39	[0.2; 1]	[41]
Δ*p*	Difference of symmetric divisions (basal to parabasal)	0	[−; −]	fixed
Δ*q*	Difference of symmetric divisions (parabasal to differentiated)	-0.012	[−1; 1]	calibrated
*ν*	Keratinocyte migration rate (mid/upper to surface layer, day^−1^)	0.4	[0.2; 1]	[41]
*μ*	Keratinocyte natural death rate (cell^−1^⋅ day^−1^)	0.67	[0.2; 1]	[41]
	***b. culture estimates (data-derived)***			
*N*_*b*_	Basal cell carrying capacity per FOV	47	[-; -]	fixed*
*ρ*_*b*_	Basal cell replication rate (day^−1^)	0.061	[0.060; 0.062]	estimated
*ρ*_*p*_	Parabasal cell replication rate (day^−1^)	0.0082	[-; -]	estimated*
Δ*p*	Difference of symmetric divisions (basal to parabasal)	≈ 0	[-0.02; 0]	estimated
Δ*q*	Difference of symmetric divisions (parabasal to differentiated)	−0.99	[-1; -0.96]	estimated
*ν*	Keratinocyte migration rate (mid/upper to surface layer, day^−1^)	0.18	[0.15; 0.21]	estimated
*μ*	Keratinocyte natural death rate (cell^−1^⋅ day^−1^)	≈ 0	[0; 0.01]	estimated

**Fig 2:**
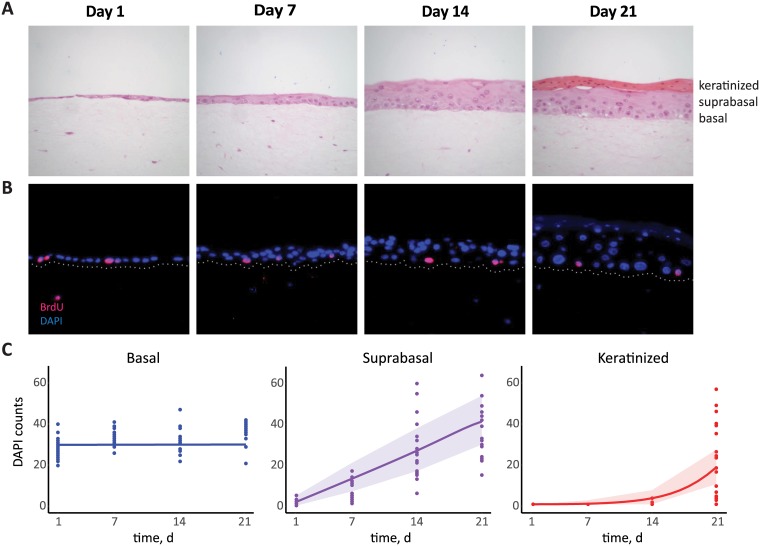
Epithelial cell growth in 3D raft cultures. A) NIKS grown from a single layer over a period of three weeks. Dark pink layer in week 3 consist of cornified cells that accumulate on the surface. B) Immunoflourescence staining: DAPI (blue) is nuclear staining for cell counting and BrdU (pink) is for identifying cells undergoing division; white dots are added to delineate basal lines. C) Data of NIKS growth over time with model fitting. Shading corresponds to 95% prediction interval, assuming the data follows a Poisson distribution.

The parameters for which we have less information are related to the fraction of cells dividing symmetrically (e.g. a parabasal cell produces two daughter parabasal cells or two differentiated cells). Existing data suggests symmetric divisions are expected to be low [42, 43]. This is further reinforced by our estimate of epithelium thickness. Histological studies calculate 26 to 28 cell layers in the vaginal epithelium depending on the stage of the menstrual cycle [44] and *in vivo* studies of the cervical epithelium count 16 to 17 layers [45]. To achieve comparable values, and assuming that the ranges of the other parameters are biologically plausible, we find that symmetric divisions must be rare. Calibrating Δ*q* ≈ −0.012 gives an epithelium ‘thickness’ of 17 layers, i.e. 17*N*_*b*_. Analytical results shown in S1 Text revealed the need for some degree of symmetric division biased towards producing differentiated cells (Δ*q* < 0). Furthermore, if we assume each layer of parabasal cells has the same number of basal cells and that the differentiated cells are half the number of cells per layer (because they are twice the size [46]), then 17*N*_*b*_ corresponds to 26 layers. Finally, we found that the mid layers, that is the differentiated, *U*_*d*_, and parabasal layers, *U*_*p*_, are larger than the basal and superficial, *U*_*s*_, layers. To obtain experimentally relevant parameter estimates, we used our model and the known parameters as priors to estimate values using original data from raft cultures of NIKS (Normal Immortal Keratinocytes) cells. The NIKS cell-line grows into a 3D epithelium structure and is commonly used as a model of cervicovaginal tissue and HPV infections, though they are known to differ from *in vivo* tissue [40]. Fig 2A and 2B show an example of NIKS cell growth into stratified form. Fig 2C shows the dynamics of the number of basal and suprabasal (non-keratinized and keratinized) cells, along with the inferred dynamics from the model. From this data (of growth from single layer to stratified) the symmetric divisions were inferred to be negligible in the basal layer but important in the parabasal layers (Table 1). This implies then that the constant basal layer assumption, and thus model 1, is appropriate for fitting organotypic culture datasets. The data constrained the replication rate of the parabasal cells, *ρ*_*p*_, to be low and the Δ*q* was estimated to be close to −1, suggesting that while the replication rate is low, nearly all parabasal divisions produce two differentiated cells which move up the column (Table 1). This, along with the higher than *in vivo* estimates for the basal replication rate, *ρ*_*b*_, is consistent with a growth phase of an epithelium growing up to homeostasis.

We performed a sensitivity analysis to explore the general behavior of the model and identify the parameters that have the largest effect on homeostasis, i.e. at a dynamic equilibrium without infection (Table 2). This showed that the total number of cells in the layers above the basal layer is mostly governed by the basal cell proliferation rate, *ρ*_*b*_. Additionally, the time for the system to reach homeostasis (which is important for repairing damaged tissues) depends on the proliferation rate of the parabasal cells (*ρ*_*p*_; S1 Text). Indeed, homeostasis is reached faster when the replication rate, *ρ*_*p*_, or the symmetric divisions of the parabasal cells, Δ*q*, are significantly higher, as found from fitting the data and the model simulations (Table 1 and not shown).

**Table 2:** Sensitivity analyses of key infection properties. For each pathogen, we show the three most important parameters with the associated partial rank correlation coefficient (PRCC) and its 95% confidence interval. Notations for parameter values are in Tables 1 and 3. Peak of infected cells is a measure of the size of the infection, peak of free-virion (or elementary bodies, EBs) load is how much progeny is released for re-seeding the infection or transmission, and day of peak is a measure for how quickly the infection grows. For effects on protection by epithelial parameters we tested: *ν*, *μ*, *ρ*_*b*_, *ρ*_*p*_, *ζ*, and *ζ*_*u*_.

**Uninfected Epithelium**			
Total number of cells	*ρ*_*b*_: 0.72 [0.68, 0.74]	Δ*q*: 0.66 [0.61, 0.70]	*ν*: -0.45 [-0.50, -0.39]
Time to homeostasis	Δ*q*: 0.62 [0.58, 0.67]	*ρ*_*p*_: -0.60 [-0.66, -0.55]	*ν*: -0.41 [-0.45, -0.35]
**wart-associated HPV**			
***effects of epithelial parameters***			
Peak of infected cells	*ρ*_*b*_: 0.98 [0.98, 0.99]	*ν*: -0.94 [-0.95, -0.93]	*μ*: -0.73 [-0.77, -0.69]
Peak of free-virion load	*ζ*: -0.96 [-0.97, -0.96]	*ρ*_*b*_: 0.94 [0.94, 0.95]	*ν*: -0.85 [-0.87, -0.83]
Duration of infection	*ζ*: 0.96 [0.95, 0.97]	*ρ*_*b*_: -0.93 [-0.94, -0.92]	*ρ*_*p*_: -0.85 [-0.87, -0.82]
Day of peak	*ζ*: 0.95 [0.95, 0.96]	*ρ*_*b*_: -0.93 [-0.94, -0.92]	*ρ*_*p*_: -0.88 [-0.90, -0.86]
***effects of infection parameters***			
Peak of infected cells	*ρ*_*a*_: 1.00 [0.99, 1.00]	*κ*: -0.84 [-0.85, -0.83]	*θ*: -0.02 [-0.09, 0.05]
Peak of free-virion load	*θ*: 0.92 [0.91, 0.93]	*ρ*_*a*_: 0.88 [0.87, 0.90]	*β*: 0.05 [-0.002, 0.11]
Duration of infection	*κ*: -0.96 [-0.97, -0.96]	*β*: 0.71 [0.67, 0.73]	*θ*: 0.71 [0.68, 0.75]
Day of peak	*κ*: -0.99 [-1.00, -0.99]	*β*: -0.49 [-0.55, -0.42]	*θ*: -0.49 [-0.55, -0.41]
**HR-HPV**			
***effects of epithelial parameters***			
Peak of infected cells	*ρ*_*b*_: 0.98 [0.97, 0.98]	*ν*: -0.94 [-0.94, -0.93]	*ζ*: -0.91 [-0.92, -0.89]
Peak of free-virion load	*ζ*: -0.97 [-0.97, -0.97]	*ρ*_*b*_: 0.95 [0.95, 0.96]	*ν*: -0.87 [-0.86, -0.85]
Duration of infection	*ζ*: 0.97 [0.96, 0.97]	*ρ*_*b*_: -0.95 [-0.96, -0.94]	*ν*_*p*_: 0.86 [0.84, 0.88]
Day of peak	*ζ*: 0.97 [0.96, 0.97]	*ρ*_*b*_: -0.95 [-0.96, -0.94]	*ν*_*p*_: 0.86 [0.84, 0.88]
***effects of infection parameters***			
Peak of infected cells	*a*_*b*_: 0.93 [0.92, 0.94]	*ρ*_*a*_: 0.91 [0.90, 0.92]	*a*_*p*_: 0.24 [0.17, 0.31]
Peak of free-virion load	*θ*: 0.87 [0.86, 0.89]	*ρ*_*a*_: 0.80 [0.78, 0.83]	*a*_*b*_: 0.77 [0.75, 0.81]
Duration of infection	*κ*: -0.95 [-0.95, -0.94]	*θ*: 0.68 [0.64, 0.71]	*β*: 0.66 [0.62, 0.70]
Day of peak	*a*_*p*_: -0.99 [-0.99, -0.99]	*κ*: -0.94 [-0.95, -0.94]	*ρ*_*a*_: -0.15 [-0.22, -0.09]
**Chlamydia**			
***effects of epithelial parameters***			
Peak of infected cells	*ρ*_*p*_: 0.95 [0.94, 0.96]	*ν*: -0.94 [-0.94, -0.93]	*ζ*_*u*_: -0.91 [-0.92, -0.90]
Peak of EBs	*ζ*_*u*_: -0.97 [-0.97, -0.96]	*ρ*_*p*_: 0.91 [0.90, 0.92]	*ν*: -0.89 [-0.91, -0.87]
Duration of infection	*ζ*_*u*_: -0.94 [-0.95, -0.93]	*ρ*_*b*_: 0.92 [0.91, 0.93]	*ν*: -0.87 [-0.89, -0.86]
Day of peak	*ζ*_*u*_: 0.94 [0.93, 0.95]	*ν*: 0.93 [0.92, 0.94]	*ρ*_*p*_: -0.91 [-0.92, -0.89]
***effects of infection parameters***			
Peak of infected cells	*β*_*u*_: 0.68 [0.64, 0.71]	*β*_*p*_: 0.53 [0.50, 0.58]	*β*_*b*_: 0.28 [0.22, 0.36]
Peak of EBs	*β*_*u*_: 0.79 [0.76, 0.81]	*η*_*u*_: -0.68 [-0.73, -0.65]	*β*_*p*_: -0.61 [-0.66, -0.57]
Duration of infection	*β*_*p*_: 0.85 [0.84, 0.87]	*f*: -0.65 [-0.69, -0.61]	*η*_*u*_: 0.37 [0.32, 0.42]
Day of peak	*β*_*u*_: -0.82 [-0.84, -0.80]	*β*_*p*_: -0.79 [-0.82, -0.77]	*η*_*u*_: -0.33 [-0.40, -0.26]

Having generated and calibrated a model for epithelial dynamics, we could then simulate infections to investigate how stratification affects important properties of the infection.

### Symptoms during infection: Disruption of homeostasis

**Fig 3:**
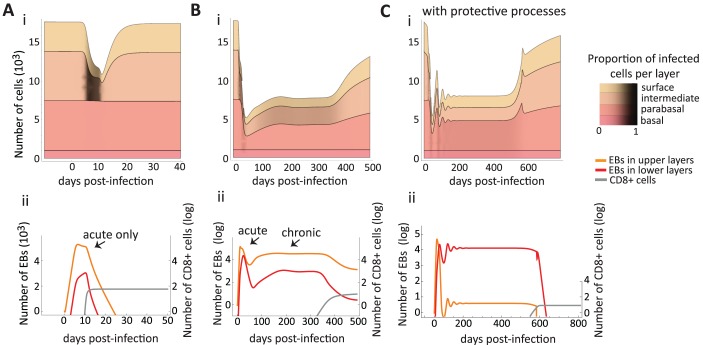
Simulated population dynamics of epithelial cells, immune effectors and bacteria in chlamydia infections. In A, the immune cell proliferation is rapid, which leads to an acute infection. In B and C, immune cells do not proliferate fast enough to clear the bacteria and the acute phase is followed by oscillations and the establishment of a chronic phase (plateau of EB density). The infection is lytic reducing the thickness of the epithelium (A, B and C) but only chronic infections manage to infect the lower layers (B and C). Parameter values are default (Table 3 and literature values in Table 1) except in A where *β*_*b*_ = 8.0 × 10^−7^ cell^−1^⋅ EB^−1^⋅ day^−1^, *β*_*p*_ = 4.0 × 10^−6^ cell^−1^⋅ EB^−1^⋅ day^−1^, *β*_*u*_ = 2.0 × 10^−5^ cell^−1^⋅ EB^−1^⋅ day^−1^, and *φ* = 0.0015 day^−1^. In C, all four epithelial protective measures happen together, i.e. *ζ*_*u*_, *ρ*_*b*_, *μ*, and *ν* all rise logistically to a threshold above default after infection to mimic the protective epithelial response (see section A.3 S1 Text, eqn 7a with *θ* = 1). Thresholds used in C: $\zeta_{u_{max}}=6\;{\text{day}}^{-1}$, $\rho_{b_{max}}=0.3\;{\text{day}}^{-1}$, *μ*_*max*_ = 0.9 cell^−1^⋅ day^−1^, and *ν*_*max*_ = 0.8 day^−1^.

Epithelial infections by both chlamydia and HPVs are heterogeneous in their clinical manifestations. Chlamydia infections can be asymptomatic or with clinical manifestations such as cervicitis [47]. The lytic nature of chlamydia infections reduces the epithelium to lower cell numbers than homeostasis, therefore affecting the integrity of the layers (Fig 3). This is consistent with the cervical erosion observed in chlamydia-driven cervicitis or in infections by other lytic pathogens such as HSV [48].

**Fig 4:**
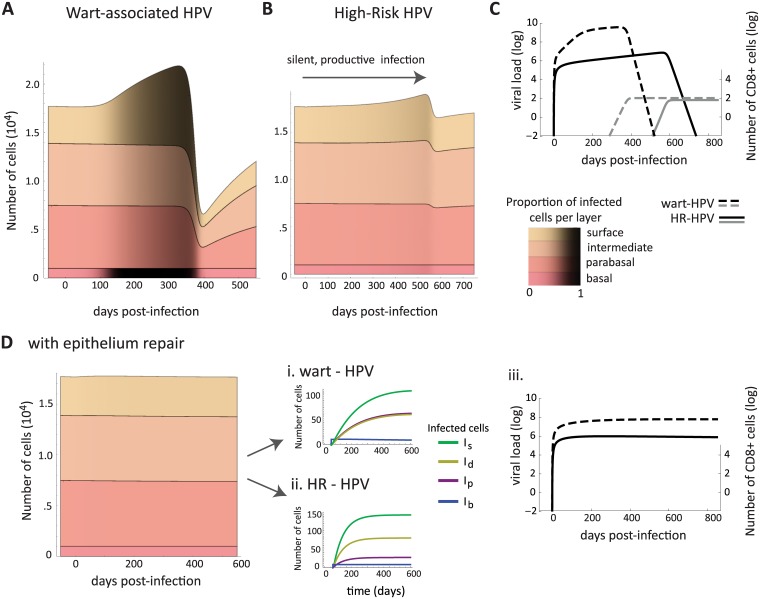
Simulated population dynamics of epithelial cells, immune effectors and free viruses in the case of HPV infections. Wart-like epithelial dynamics in a wart-associated HPV infection **(A)** and a slow growing high-risk (HR) HPV infection that spontaneously regresses **(B)**. The black shading shows the proportion of infected cells in each layer. **(C)** Dynamics of virus load (black) and the density of immune effectors (gray) for wart-associated HPV (dashed line) and HR-HPV (full line) infections. Immune cells start to proliferate upon infection but their number remains below −2 log for several months. **(D)** Simulated scenario where the infection is inoculated with few cells and the microabrasion repairs quickly: this results in both wart-associated and HR types causing asymptomatic infections. Here, the model predicts that infections with HR types have more infected cells, due to their higher proliferative properties, but wart-associated types produce more virions (i, ii, iii), and both infections can last for years, if stochasticity or the innate response do not clear them (i, ii). Parameter values are default (Tables 1 and 3) and infection models are 3. Infection rates of both wart-associated and HR HPVs are identical (*β* = 10^−10^) and all infections begin with 10 infected basal cells. In (D) the infection rate, *β*, decays to zero in 10 days (*b* = −0.5) to mimic tissue repair (see section A.3 in S1 Text for details).

Several HPVs have been found to be associated in wart-like lesions, which are substantive cell overgrowth above homeostasis levels. Among the mucosal *α*-genus HPVs, HPV6 and HPV11 often (though not always) generate papillary lesions or warts. In cutaneous stratified squamous epithelia several HPVs are associated to warts (e.g. species 2 and 4 of the *α*-genus and the types of the *μ*- and *ν*-papillomaviruses) in various locations, such as the feet and hands [49]. Conversely, HR-HPV types cause flat lesions (yet with a thickening of the epithelium) [37]. How differences between HPV types translate into this observed diversity of clinical manifestations in the epithelium is not always clear. What is clear is that HR-HPVs have stronger cell transforming properties than low-risk (LR) and wart-associated HPVs [37]. The epithelium model allowed us to identify conditions that lead to wart-like manifestations. When assuming that there can be rare events of new virions entering the basal layer (e.g. due to immunosuppression and cytokines loosening epithelial junctions) and that wart-associated types do not drive cell proliferation in lower layers [37], we find that they must either have higher burst sizes (produce more virions per cell) than HR-HPV types or be better at driving differentiated cells back into S-phase in the upper layers (*ρ*_*a*_ and *θ* control the peak of infected cells in Table 2). Burst size, *θ*, also controls how quickly the number of infected cells increases, as does the infection rate, *β*. This explains why simulations of wart-associated HPVs with higher burst sizes are more effective at reaching basal cells, as illustrated by the differences in shading of basal layers between Fig 4A and 4B. Epidemiological studies that directly compare viral loads of LR vs. HR genital HPVs are needed, however, wart-associated HPVs (mucosal or cutaneous) have higher viral loads in warts than other HPVs [49].

HR-HPVs have enhanced E6 and E7 oncoprotein effects in the lower layers [37]. In spite of this increase in epithelial cell division rate their infections are flat, slow growing, and are often clinically indistinguishable from a normal epithelium for many months. For this to occur, we find that the extra proliferation in the basal, *a*_*b*_, and upper layers, *ρ*_*a*_, and the type’s burst size, *θ*, must be kept low (Table 2 and Fig 4B). This implies HR-HPVs would be less ‘productive’ (shed less virions) than wart-associated HPV during an infection of the same duration (Fig 4C). If HR-HPVs were to have low burst sizes but high oncoprotein-driven proliferation in the lower layers, then their infections would be wart-like (S1 Text, S2 Fig). Thus, to maintain flat lesions the strong HR oncogenes need to be down-regulated. A simulated representation of silent, productive HR-HPV infection is shown in Fig 4B.

For an infection to be sufficiently disruptive to generate a visible manifestation, both the size of inoculum (number of cells infected initially) and how quickly the microabrasion closes from repair appear to matter. For instance, the wart-like overgrowth of cells in Fig 4A can be created either by a small inoculum with slow repair or by a large inoculum and fast repair. When microabrasions close quickly (within a few days) and only a small number of cells are infected initially, both HR and wart-associated types do not cause any visible disruption to homeostasis Fig 4D. Clinically, these infections would be asymptomatic with normal cytology and would likely only be detectable using PCR methods.

Finally, we compared our HPV results with a non-stratified model of HPV infection (see S1 Text) and find that it is unable to reproduce the features associated with HR and non-HR HPV infections if using the same biologically constrained parameter ranges (see S4 and S5 Figs).

### Infection duration and persistence

For some parameter combinations the kinetics of chlamydia infections had an acute phase only (Fig 3A), as have been observed in guinea pigs and other animal models [18]. We obtain this qualitative pattern most readily when the infection rates are the same for all layers or when the lower layers are difficult to infect (for instance due to the reduced permeability down the epithelium column [12]) and the population of immune effectors grows rapidly. From the sensitivity analysis, duration is longer when the EBs can infect the lower layers, *η*_*u*_, and shortened when the cell recovery rate is high (Table 2).

We also found an acute phase can be followed by a chronic phase, where a pathogen load stabilizes to a set point value (Fig 3B). How quickly a chronic phase is reached depended on chlamydia’s infection rates of the various layers. Generally, infection rates had to be low to achieve the chronic phase (because if too high then the bacteria burn through the epithelium and its population crashes). Additionally, if the layers are differentially infected by chlamydia (i.e. *β*_*b*_ < *β*_*p*_ < *β*_*u*_), then the chronic phase is reached earlier (see S3 Fig).

In contrast, long-lasting wart-associated and HR HPV infections did not exhibit an acute and a chronic phase in our model. Instead, they persisted by monotonically reaching an equilibrium (e.g. Fig 4Di and 4Dii). Also, for both HPVs, the immunity killing rate, *κ*, was the most important factor in determining infection duration (Table 2). With more antigen in the lower layers to detect, the efficiency of immune killing (*κ*) becomes important for determining duration of infection, speed of growth and size of infected cell accumulation (Table 2).

Finally, while *Chlamydia* and HPVs can cause either acute or chronic infections [51], our model showed that a clinically detected chronic state is achieved through different underlying dynamic patterns for each pathogen.

### Protective effect of epithelial dynamics against infection

Upon infection, epithelia exhibit defense mechanisms such as increasing mucus flow, tightening the packing of cells, migration to the surface [52] and increasing proliferation (promoted by Interleukin-22 cytokines [53–55]). We varied epithelial parameters from their homeostasis value to investigate in detail the effect of such mechanisms on various measures of infection using our infection models for HPV and chlamydia (models 3, 4 and sensitivity analyses in Table 2).

We found some mechanisms had similar effect on both HPVs and chlamydia. First, increasing upward migration of epithelial cells, *ν*, reduced the maximum pathogen load reached during the infection (Table 2). Second, mucus trapping, *ζ*, delayed the peak and the duration (although it played a bigger role in decreasing the peak of infection for chlamydia than for HPV). And finally, for all infections, increasing basal or parabasal cell proliferation, *ρ*_*b*_ and *ρ*_*p*_, scored high in affecting all the infection measures, e.g. size of peak or duration (‘effects of epithelial parameters’ in Table 2). However, a pathogen-specific effect was that increasing basal proliferation, *ρ*_*b*_, of uninfected cells decreases the time to clear HPVs but not chlamydia. Together, this suggests that epithelial cell features themselves play an important role in infection dynamics and outcomes.

## Discussion

Epithelial infections are a major public health burden, and, in particular, STIs are on the rise causing a worldwide concern [1, 9, 56]. Quantitative models, both experimental and mathematical, are essential in developing our understanding of these infections. As for systemic (and virulent) infections such as HIV and HCV, mathematical models have been developed to predict and analyze the kinetics of epithelial infections. Here, we show that to understand the kinetics of epithelial infections, it is essential to account for the stratified structure of the epithelium, a property that is absent from most models. We illustrated how such a general framework can be combined with 3D cell culture data to estimate key parameters and how it can generate relevant insights regarding the course of epithelial infections.

### Dynamical implications of ecological features

The rate of basal cell proliferation had a strong effect on the homeostasis of both uninfected and infected epithelia, which suggests an ecological ‘bottom-up controlled’ system [57, 58], analogous to those found in free-living food webs. These bottom-up effects are more apparent if we consider that basal cell replication is strongly determined by the resources that are available in the basal lamina, such as growth factor. While we did not explicitly model the resources of the basal layer (it is implicit in the basal proliferation rates), the growth of the cells in the experimental set-up does depend on concentration and temporo-spatial distribution of growth factors, impacting epithelial thickness and proliferation rates. Therefore, this ecological insight of bottom-up driven systems, could be tested more formally in experimental systems by monitoring resource concentrations.

The key role of bottom-up control is further supported by our finding that accelerating basal cell proliferation, as a response to infection [53, 54], affected all infection measures (e.g. time of peak, total load, duration). This infection response, then, can have a strong effect on the severity and duration of infections. However, using the same response mechanism might be differentially effective depending on the infection strategy of the pathogen. For instance, we found that increasing cell proliferation did not shorten the infection of chlamydia. This is probably because proliferation increases the number of uninfected epithelial cells in the upper layers which, for chlamydia, means more ‘resources’.

Pathogens can have different tropisms for the various cell phenotypes of the stratified epithelium. For instance, EBV more readily infects and replicates in differentiated cells of the upper/mid layers [59], whereas HPV infects the basal layer to establish an infection [37]. We hypothesized that this should impact how effective protective processes (e.g. increased mucus production) of the epithelium are against them. In chlamydia, where the pathogen infects all cell types equally well, we found that tight packing (i.e. epithelial permeability) mattered to the pathology at the site. The speed at which the epithelium shrank and the stability of the infection system (how quickly it can reach chronic phase) depended on how well the bacteria could access cells down the column. If the bacteria were able to infect the bottom of the column quickly, that led to a population crash due to the lack of resources. On the contrary, and somehow unexpectedly, less epithelial permeability stabilized the infection that then lasted much longer and exhibited a clear chronic phase. This stabilizing effect is also observed in ecological systems when one stage is invulnerable to attack, i.e. a stage refugia [32, 60]. For instance, a parasitic wasp was introduced as a biological control of red scales (a common plant pest). It successfully controlled the red scales because one of the mature stage of the red scales was not vulnerable to attack [32]. Such effects from decreasing permeability (protecting the basal replicative stage) would have implications in the context of treatments that bolster cell adhesion and require testing experimentally.

Considering pathogens with contrasted life-histories allowed us to identify how similar infection outcomes arise. In the case of chlamydia, the interaction between free-form chlamydia and its infection rates of the various stages drove the chronic phase, but although the activation of the immune response through the same feedback ultimately led to clearance, this feedback affected several infection features. In contrast, HR-HPV persistence was achieved via a slow growth strategy that delays clearance by decreasing the negative dynamical feedback involving the immune system (i.e. faster growth implies faster immune detection and clearance). Indeed, HPV types appear to evade, or counteract, these immune responses differently. In particular, viral protein E6 of various HPV types differ in their many cellular binding partners resulting in a variety of effects on host processes [61]. We found that the difference between HPV-induced genital warts and epithelial lesions depended most on the number of virions an infected cell releases upon death (or ‘burst size’) and the initial size of inoculum; suggesting that more productive viruses are better colonizers. A ‘colonization’ strategy (in ecology ‘r strategy’) appeared to have a cost for the virus because infecting the basal layer of the epithelium triggers the immune response. While more sites are colonized, each site is exploited less optimally. Another feature that was mediated through the immune response feedback was that mucus trapping delayed the peak of the infection (i.e. the decreased progeny of bacteria and viruses meant less antigen and thus slower immunity detection).

To compare our results to HPV epidemiological studies of acute HPV infections, we see that the model creates underlying patterns (e.g. viral load Fig 4C) that could be looked for using prospective studies of HPV infections with normal cytology. Study designs with dense sampling (with visits every 3 or less months) are best for capturing the dynamics of these infections, particularly for the exponential increase and decay of viral loads. The majority of HPV prospective studies are of persistent infections and with advancing cytological abnormality but there are exceptions. For instance, Marks et al. sampled young women with HPV16 infections every 3 months and found that a greater than 2 log decrease in viral load was associated to clearance and a single viral load measure could not predict clearance [62]. The HR-HPV viral load dynamics from our model (Fig 4C) can provide possible underlying explanations and our exponential decrease is consistent with the decrease found by this epidemiological study. Though, sampling once would not give enough information as to whether the infection is increasing or decreasing at a given point. Consecutive viral load measures, then, are more appropriate to estimate clearance or persistence [63].

The effect of stage-structure on infection dynamics can be interpreted in light of earlier results from ecology or epidemiology. For instance, in epidemiology, it is known that the more a general population of infected host is subdivided into classes, the more rapid the growth rate of the epidemic is and the shorter it lasts [64]. Our model bears even more parallels with age structured models in epidemiology where the age groups of the host population are explicitly considered. In many of these models, children tend to be key to the spread of epidemics [64], a result that echoes the bottom-up effects we identify. However, the driving forces in the two models are different: in our model it is due to the fact that basal cells are the ones replicating, whereas in epidemiology it is usually driven by longer lasting acquired immunity at higher ages.

### Perspectives

Spatial structure is a natural extension of our model that could be investigated further. Here, the different cell populations partly capture the vertical structure. A specific consequence of not including space is that the immune system effects are more homogeneous than in reality, where more resident immune cells are present in the lower layers. The assumption of well-mixed populations holds best when the model represents a portion of the squamous epithelium (rather than, for instance, the whole cervix). In the case of patchy infections like HPV, a metapopulation modeling approach may be more appropriate (e.g. [22, 38]) or a full spatial model [21]. We chose not to include space since much of the experimental data available on these systems is not spatial. Instead most are cell population counts from immunofluorescence or flow cytometry techniques. Several mathematical modeling methods, such as agent-based models, are available to study spatial aspects of infections, particularly cell-to-cell spread [65]. These should be of interest to those studying chlaymdia infections. Even though HPVs have not been found to spread cell-to-cell like other viruses [66], studying the spatial aspects of their infections should most certainly still be considered in future studies.

Introducing stochastic aspects in epithelial dynamics have recently refueled the discussion on the determinants of HPV clearance [23]. In general, considering stochastic dynamics could matter most when pathogen populations approach low-levels (i.e. very few infected cells or small loads). For instance, our finding that mucus trapping can delay the peak and the duration of infections could interact with stochasticity. This is similarly true for infections started with a small inoculum, very rapid abrasion closure, and rapid repair with small inoculum. These processes keep the pathogen populations sizes down and thus, as seen in ecological systems, stochasticity should play a larger role in extinction. As for the spatial structure, it is important to stress that there often is little data on the initial stage of the infections, when the pathogen is rare.

Many previous works have used simplified descriptions of the immune response in a similar fashion as we have chosen to model here [15, 27]. Models with simplified immunity usually ask conceptual questions or are used to infer parameter values from data with few measured cell types (e.g. only counting CD8^+^ and CD4^+^ T cells). Future work interested in specific questions that are immune related, for instance comparing the respective roles of innate and adaptive immunity in clearance, could benefit from more detailed descriptions of immune effectors. In particular, expression of cytokines are interesting as they are important in the epithelium’s part in innate immunity [52].

Our model does not attempt to capture the progression stages that HPVs can cause in persisting infections. To appropriately model these changes would require several adjustments, including that cell proliferation of infected cells and probabilities of symmetric divisions become time variant. Indeed, our model can be adapted to study other oncoviruses that infect the epithelium, where future studies can consider the contexts of immune evasion and cellular transformation driven by oncogenes [37]. In addition, there is increased interest in how epithelial cell dynamics (e.g. cell competition, mechanisms to maintain homeostasis and repair) interact with our knowledge of how tumor viruses alter cellular programing, in particular changing balanced cell fate ratios, skewing squamous differentiation toward a proliferative phenotype [67]. New modeling methods will require possible evolutionary approaches of cell phenotypes emerging over time.

In many ways, the simultaneous infection of a host by different pathogen strains or even species is the rule rather than the exception [68]. Of particular interest is how different pathogens or strains interact inside a host and how this affects the course of the infection. For instance, HPV infections are often of multiple HPV types and as lesions progress to cancer there is clonal-selection, usually leading to a single type as the main driver of the tumor [69]. One straightforward extension of this model would be to investigate coinfections between pathogens with similar cell tropisms (e.g. chlamydia and EBV) or pathogens that differ in their life-cycles. Our model could consider both infections at once or be adapted to study organotypic models that include multiple pathogen infections (e.g. HSV, EBV and HPV coinfecting the same tissues and cells [70]) or the effects of the resident microbiota.

Finally, opening a dialogue between mathematical modeling and experimental data generates new hypotheses to test. One of the clearest illustrations of this is our result that burst size differences appear as the most parsimonious explanation to explain symptom differences between wart-causing and lesion-causing HPV infections. Technological improvements in clinical and experimental techniques also allow us to test more subtle predictions. Testing hypotheses generated by the model will allow us to move forward by validating the model assumptions that are consistent with the data and rejecting the others. This will allow us to increase the model complexity and test more elaborate predictions. We hope to inspire experimental studies on infections of stratified epithelia to focus more on dynamics and time series approaches (including mathematics) to better understand these varied and broadly impacting pathogens.

## Materials and methods

### Ethics statement

The Thunder Bay Regional Health Research Institute’s Biosafety Committee approved all research involving NIKS cell line cultures. The NIKS cell line [40] was obtained from Dr. Paul Lambert, McArdle Laboratory for Cancer Research, University of Wisconsin.

### Cell culture data

Organotypic culture growing techniques used here have already been described in detail elsewhere [71, 72]. Original experiments were performed to obtain time series data with sufficient replicates for model fitting. Three independent experiments were performed, with rafts harvested at one-week intervals (0, 1, 2, and 3 weeks) starting the day after lifting them to an air-liquid interface. From a total of 12 formalin-fixed, paraffin-embedded (FFPE) rafts, 48 tissue slices were imaged using fluorescence microscopy (DAPI staining for cell nuclei) and resulted in 3 Fields of View (FOV) per slice (*n* = 144). Counts in each FOV were done semi-automatized using ImageJ cell counting software.

### Epithelial model

The uninfected epithelial model consists of 4 cell populations of distinct phenotypes to capture epithelial structure (Fig 1): basal cells (assumed to have a constant population size, *U*_*b*_ = *N*_*b*_, as cells that move up are replaced immediately), parabasal cells (with population size *U*_*p*_), differentiated cells of the mid and upper layers (with population size *U*_*d*_) and of the surface layer (with population size *U*_*s*_). Since we are interested in cervicovaginal infections of non-keratinized squamous epithelia, we assume the top layer of keratinocytes are close to death and are shed from the surface as they die. The cell population dynamics are captured by three ordinary differential equations (ODE):

$$\begin{array}{c} \begin{array}{cc} \dot{U_{s}} & =\nu \;U_{d}-\mu \;U_{s}\\ \dot{U_{d}} & =\rho_{p}(1-\Delta q)U_{p}-\nu \;U_{d}\\ \dot{U_{p}} & =\rho_{b}(1-\Delta p)U_{b}+\rho_{p}\;\Delta q\;U_{p} \end{array} \end{array}$$ (1)

Dots above the variables indicate time derivatives. Basal cells proliferate at a rate *ρ*_*b*_, giving rise to parabasal cells which in turn proliferate at a rate *ρ*_*p*_, while entering the mid and upper layers of the squamous column (Eq 1). These cells are differentiated and migrate up to the surface layer at a rate *ν*. Mature keratinocytes die at the surface of the epithelium at a rate *μ*. There is an overlap between cell phenotype and spatial structure since an epithelial cell moves up the stages as it ages (Fig 1).

When modeling stem cell divisions, we follow earlier studies [23, 73] and introduce the fraction of basal cell divisions that are symmetric and give rise to two basal cells, *p*_1_, and the fraction that creates two parabasal daughter cells is *p*_2_. Note that *q*_1_ and *q*_2_ are the parabasal equivalent terms (see Fig 1). The generation of parabasal cells from basal cells is found by 2 *p*_2_ + (1 − *p*_1_ − *p*_2_) which we simplify to 1 − Δ*p* by assuming Δ*p* = *p*_1_ − *p*_2_ and the equivalent of this for the generation of differentiated cells is Δ*q* = *q*_1_ − *q*_2_ in equation system 1. We considered distinct probabilities of divisions for the two layers (*p*s and *q*s), even though both the basal and parabasal layers are mostly made up of the same transit-amplifying cells, because the basal layer also contains stem cells which can divide in an unlimited fashion [74]. Thus, the two layers should have distinct properties. Finally, the assumption that the basal layer is constant implies that we must assume Δ*p* = 0 in order for the basal layer to neither grow nor shrink. However, we maintain this structure of the model because Δ*p* would be needed if one were to either relax the assumption of a constant basal layers (e.g. when studying a growing epithelium, as in organotypic cultures) or when it is infected (e.g. HPV infections might alter this parameter and make *p*_1_ divisions more frequent [67]; though we do not address this feature of HPV infection directly).

We chose to not include the stochastic nature of these cell divisions, as it has been considered previously [23, 73], and we were interested in understanding deterministic behaviors of the system, such as active repair or active changes to cell ratios. All the variables and parameters used are summarized in Fig 1 and Table 1. Finally, the model is sufficiently general that it can represent different kinds of stratified epithelia, including keratinized and non-keratinized squamous epithelia.

To calibrate parameters (Table 1), we initially relied on a study from 1970 that used *in vivo* autoradiography techniques to calculate the mean cell cycle time for epithelial cells in cervical and vaginal tissues [41]. They found that basal cells have a relatively slow cycle of approximately 33 days and that 1.14% of these cells are synthesizing DNA at a given time point. Parabasal cells have a much shorter cell cycle (2.6 days) and 14.25% of these cells are synthesizing DNA. Differentiated cells do not divide and have a life expectancy of 4 days (Table 1). A detailed analytical analysis of this uninfected model can be found in the S1 Text.

For fitting raft cell culture data, we did not want to assume *a priori* that the basal layer starts off as a constant, especially since in the experiments the tissue is grown-up from a single layer cells. So we used a variation of our model by assuming the basal layer was not constant but rather followed this equation:

$$\begin{array}{c} \dot{U_{b}}=\rho_{b}\;U_{b}\;\Delta p\;(1-\frac{U_{b}}{N_{b}}). \end{array}$$ (2)

Here we assume the basal layer (cells that are touching the basal lamina) are growing until they reach a maximum capacity, *N*_*b*_, and Δ*p* is not assumed to be zero. There are other changes from the previous model: *U*_*s*_ now represents the surface cells that are keratinized, and since the *U*_*p*_ and *U*_*d*_ cells cannot be distinguished experimentally we summed these two variables for fitting the ‘suprabasal’ cells counted in the experiment.

### Infection models

**Fig 5:**
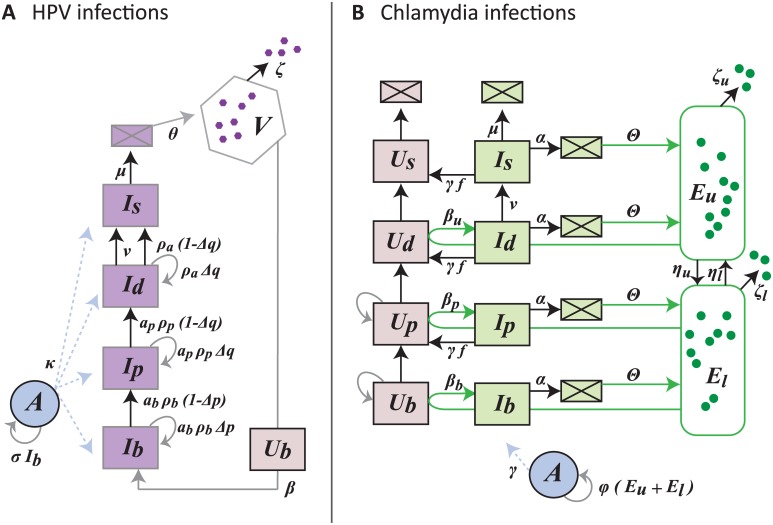
Flow diagram of the infection models for HPV (A) and chlamydia (B). HPV virions, *V*, only infect uninfected basal cells, *U*_*b*_, to become basal infected cells, *I*_*b*_. Since HPV is non-lytic, infected cells follow the typical epithelial life-cycle up to the surface passing through different life stages (parbasal *I*_*p*_, differentiated *I*_*d*_, differentiated at the surface *I*_*s*_). Model 3. In the case of *C. trachomatis*, the elementary bodies, EBs, start the infection by infecting uninfected cells in the upper layers (*β*_*u*_ *U*_*d*_ *E*_*u*_ creates *I*_*d*_). The EB populations start in the upper layers, *E*_*u*_, and then migrate down, *η*_*u*_, into the lower layers, *E*_*l*_. As EBs migrate down layers they enter uninfected cells (*U*_*b*_ and *U*_*p*_) and create infected cells (*I*_*b*_ and *I*_*p*_) which die at rate *α* (boxes with square represent dead cells). The host immune response, *A*, is activated by infected basal cells in the case of HPV and all EBs in the case of *C. trachomatis*. Model 4. Note that for wart-associated HPV infections *ρ*_*a*_ = 0 and *α*_*b*_ = *α*_*p*_ = 1. See Table 3 for parameter descriptions and estimates.

Modeling infections of the stratified epithelium requires adding populations of free-forms of the pathogens, infected cells and immune cells. See Fig 5 for the schematics of the models and Table 3 for the parameter estimates.

**Table 3:** Parameter descriptions for infection models (equations systems 3 and 4), default values and biologically realistic ranges. Parameters value that were chosen for the results to be biologically consistent are indicated by ‘calibrated’. Parameter values that can be set arbitrarily without affecting the results qualitatively are referred to as ‘fixed’. Parameters varied without any a priori assumption are indicated by ‘free’.

		Default	Range	Ref
	**HPV models**			
*β*	Infection rate of HPV virions (cell^−1^⋅ virion^−1^⋅ day^−1^)	10^−10^	[10^−15^; 10^−5^]	fixed
*ρ*_*a*_	Replication in upper layers driven by wart-associated and HR HPV (day^−1^)	0.78	[0; 5]	[37]
*a*_*b*_	Multiplicative replication factor by HR-HPV in basal cells	1.5	[1; 10]	[37]
*a*_*p*_	Multiplicative replication factor by HR-HPV in parabasal cells	2.0	[1; 10]	[37]
*θ*	Differentiated keratinocyte wart-associated HPV-infected burst size (day^−1^)	10^6^	[10^2^; 10^8^]	[63]
	Differentiated keratinocyte HR-HPV-infected burst size (day^−1^)	10^4^	[10^2^; 10^8^]	[63]
*ζ*	Virion clearance rate by mucus (day^−1^)	1.18	[0.2; 3]	[18, 19]
*κ*	Removal rate by adaptive response (day^−1^)	0.0024	[0; 0.1]	fixed
*σ*	Adaptive response growth rate in HPV infection (HR 0.001)	0.0001	${\mathbb{R}}^{+}$	calibrated
	**Chlamydia model**			
*β*_*b*_	Infection rate of EBs in basal layer (cell^−1^⋅ EB^−1^⋅ day^−1^)	3.0x10^−6^	[0; 2.31]	[17–19]
*β*_*p*_	Infection rate of EBs in parabasal layer (cell^−1^⋅ EB^−1^⋅ day^−1^)	5.0x10^−6^	[0; 2.31]	[17–19]
*β*_*u*_	Infection rate of EBs in upper layers (cell^−1^⋅ EB^−1^⋅ day^−1^)	1.0x10^−5^	[0; 2.31]	[17–19]
*α*	Chlamydia’s killing rate of infected cell (day^−1^)	0.6	[0.01; 0.6]	[16, 17, 17–19]
Θ	Chlamydia-infected cell burst size (day^−1^)	200	[200; 500]	[16, 17]
*ζ*_*u*_	EB clearance rate by mucus (day^−1^)	2	[0.01; 10]	[18, 19]
*ζ*_*p*_	EB clearance rate by macrophages (day^−1^)	4	[0.01; 10]	[18, 19]
*η*_*u*_	EB migration rate down the epithelial column (day^−1^)	0.005	[0; 1]	free
*η*_*l*_	EB migration rate up the epithelial column (day^−1^)	0.001	[0; 1]	free
*f*	Fraction of removed infected cells that recover (day^−1^)	0.6	[0; 1]	free
*γ*	Removal rate by adaptive response (day^−1^)	0.2	[10^−4^; 0.5]	[17–19]
*φ*	Adaptive response growth rate in chlamydia infection	0.0001	${\mathbb{R}}^{+}$	calibrated

#### Human papillomaviruses

To establish an infection HPV virions can only infect basal cells because HPV capsids need to bind to the epithelial basement membrane to undergo conformational changes that allow cell entry [75]. HPV infections are non-lytic, thus infected basal cells, *I*_*b*_, follow their life-cycle move up the epithelium column, from parabasal, *I*_*p*_, to differentiated in the mid layers *I*_*d*_ and surface layers, *I*_*s*_. When the infected cells on the surface, *I*_*s*_, die they release the virions they contain, *V* (Fig 5A). As in the uninfected model, we assume that the total number of basal cells is constant, *N*_*b*_, but we distinguish between infected, *I*_*b*_, and uninfected basal cells (*N*_*b*_ − *I*_*b*_). In the upper layers the HPV oncogenes can drive some cell-cycle re-entry in order to promote its genome amplification [37]. As infected cells follow their life cycle up the epithelium column, the number of viral copies in the cells increases (from approximately 10–100 copies per basal cell to 10^3^–10^6^ in the upper layers [63]).

The following set of ordinary differential equations represent HPV infection dynamics:

$$\begin{array}{c} \begin{array}{cc} \frac{\text{d}V}{\text{d}t} & =\mu \;\theta \;I_{s}-\zeta \;V\\ \frac{\text{d}I_{s}}{\text{d}t} & =\rho_{a}(1-\Delta q)I_{d}+\nu \;I_{d}-(\mu +\kappa_{s}\;A)I_{s}\\ \frac{\text{d}I_{d}}{\text{d}t} & =a_{p}\;\rho_{p}(1-\Delta q)I_{p}+(\rho_{a}\;\Delta q-\nu -\kappa_{d}\;A)I_{d}\\ \frac{\text{d}I_{p}}{\text{d}t} & =a_{b}\;\rho_{b}(1-\Delta p)I_{b}+(a_{p}\;\rho_{p}\;\Delta q-\kappa_{p}\;A)I_{p}\\ \frac{\text{d}I_{b}}{\text{d}t} & =\beta \;V(N_{b}-I_{b})+(a_{b}\;\rho_{b}\;\Delta p-\kappa_{b}\;A)I_{b}\\ \frac{\text{d}A}{\text{d}t} & =\sigma \;I_{b}\;A \end{array} \end{array}$$ (3)

Most of the parameters are identical to that of the uninfected model (model system 1). Additional parameters that relate to specific infection processes are: the infection rate of basal cells by the virus, *β*, the ‘burst size’ of infected keratinocytes *θ* (how many virions are released per cell), and the clearance rate of free virions at the surface of the epithelium which is mostly due to mucus flow, *ζ*. To capture the increased cell division driven by HPV of infected basal and parabasal cells are the multiplicative factors *a*_*b*_ and *a*_*p*_, respectively; while in the upper layer HPVs cause cell proliferation of differentiated cells that otherwise would not happen, denoted *ρ*_*a*_. We assume that both wart-associated and high-risk types drive the cell proliferation in upper layer at the same rate, *ρ*_*a*_ (Table 3). High-risk HPVs are the most oncogenic and their oncogenes E6 and E7 give them the ability to force basal and parabasal cells to re-enter S-phase with the aim to have access to the cell’s replication machinery [37]. This process thereby creates more infected cells. Low-risk HPV genotypes have oncogenes with less transformative properties than the HR types and thus only drive cell proliferation in the upper layers for genome amplification but not in the lower layers. Therefore, to model wart-associated type infections we set *ρ*_*a*_ = 0 and *α*_*b*_ = *α*_*p*_ = 1 to capture these differences in cell transformation properties.

Here, our description of the immune response is intentionally simplistic. Following earlier studies (e.g. [27]), we model only one population of immune effectors as a generic adaptive immune population (typically CD8^+^ lymphocytes), *A*. The proliferation rate of immune effectors is *σ*, and the killing rates by immune effectors are considered layer specific, with *κ*_*b*_, *κ*_*p*_, and *κ*_*d*_, corresponding to their respective layer. This layer-specific assumption should be chosen when considering that immune cells come up from the dermis and thus are less numerous in the higher layers of the epithelium. However, we chose for simplicity to assume all *κ*s were the same (Table 3). Since our overall goal was to develop a generic model for infections of the stratified epithelium, tailoring the immune response was beyond the scope of this aim.

Note that the equations of ODE system 3 are run in combination with the uninfected cells Eq 1. The only adjustment made to system 1 is that the *U*_*b*_ in the parabasal layer equation is replaced with *N*_*b*_ − *I*_*b*_ the number of uninfected basal cells.

#### Chlamydia

Even though *Chlamydia trachomatis* also infects the cervix and is an obligate intracellular bacteria, its life-cycle is quite different from that of HPV’s. Chlamydia must get past dying cells in the top layer to reach living cells in the upper layer. Once the transmissible free-forms of the bacteria, the elementary bodies (EB), are taken into the cell via a membrane-bound vacuole, they transform into reticulate bodies (RB), which undergo 8 to 12 rounds of replication before turning back into EB. After approximately 30 to 84 hours post-infection the cell bursts to release the EBs [39].

Using existing models of chlamydia infection [16, 18] to modify our main model for epithelial dynamics (1), we derive the following set of equations to capture chlamydia within-host dynamics:

$$\begin{array}{c} \begin{array}{cc} \frac{\text{d}E_{u}}{\text{d}t}= & \;\Theta \;\alpha (I_{d}+I_{s})-\zeta_{u}\;E_{u}+\eta_{l}\;E_{l}-\eta_{u}\;E_{u}\\ \frac{\text{d}E_{l}}{\text{d}t}= & \;\Theta \;\alpha (I_{b}+I_{p})-\zeta_{l}\;E_{l}-\eta_{l}\;E_{l}+\eta_{u}\;E_{u}\\ \frac{\text{d}I_{s}}{\text{d}t}= & \;\nu \;I_{d}-(\alpha +\mu +\gamma A)I_{s}\\ \frac{\text{d}I_{d}}{\text{d}t}= & \;\beta_{u}\;E_{u}\;U_{d}-(\alpha +\nu +\gamma A)I_{d}\\ \frac{\text{d}I_{p}}{\text{d}t}= & \;\beta_{p}\;E_{l}\;U_{p}-(\alpha +\gamma A)I_{p}\\ \frac{\text{d}I_{b}}{\text{d}t}= & \;\beta_{b}\;E_{l}(N_{b}-I_{b})-(\alpha +\gamma A)I_{b}\\ \frac{\text{d}U_{s}}{\text{d}t}= & \;\nu \;U_{d}-\mu \;U_{s}+f\;\gamma \;I_{s}\;A\\ \frac{\text{d}U_{d}}{\text{d}t}= & \;\rho_{p}(1-\Delta q)U_{p}-(\nu +\beta_{u}\;E_{u})U_{d}+f\;\gamma \;I_{d}\;A\\ \frac{\text{d}U_{p}}{\text{d}t}= & \;\rho_{b}(1-\Delta p)(N_{b}-I_{b})\\ & +(\rho_{p}\;\Delta q-\beta_{p}\;E_{l})U_{p}+f\;\gamma \;I_{p}\;A\\ \frac{\text{d}A}{\text{d}t}= & \;\varphi (E_{u}+E_{l})A \end{array} \end{array}$$ (4)

In addition to the populations of uninfected (*U*_*p*_, *U*_*d*_, *U*_*s*_) and infected cells (*I*_*b*_, *I*_*p*_, *I*_*d*_, *I*_*s*_) of each respective layer (basal, *b*, parabasal, *p*, mid, *d*, and surface, *s*), we now have equations for the EBs in the mid/surface layers, *E*_*u*_, and in the basal/parabasal layers, *E*_*l*_. Infection of the cell kills it at a rate *α* and each cell releases Θ EBs upon dying (the ‘burst size’). EBs are cleared at rates *ζ*_*u*_ and *ζ*_*p*_. The clearance rate of EBs in the upper layers is mainly due to being trapped in the mucus of the surface. In the basal/parabasal layers, the EBs are cleared by the activity of innate effectors, especially resident macrophages [39]. We, therefore, assume *ζ*_*p*_ > *ζ*_*u*_. Finally, EBs can ‘migrate’ down and up the epithelium at rates *η*_*u*_ and *η*_*l*_ respectively.

All cells except surface keratinocytes (already near death), *I*_*s*_, can be infected by EBs at rates *β*_*b*_ for the basals, *β*_*p*_ for parabasals, and *β*_*u*_ for mid/upper layers. The increasing number of intracellular junctions down the epithelium column and the effect of space decreases susceptibility to infection from the upper layers down to the basal layers, hence we assume *β*_*u*_ > *β*_*p*_ > *β*_*b*_. Infected keratinocytes (*I*_*s*_ and *I*_*d*_) have enough time to migrate towards the surface of the epithelium. The immune effectors, *A* replicate at a rate *φ*. Finally, infected cells interact with immune effectors, *A*, at a rate *γ*. The outcome of this interaction is either cell death or cell recovery (the fraction of removed by recovery is *f*). Therefore, the populations of uninfected cells can be enriched by cell recovery upon the action of the immune response, *fγA* term. Natural clearance of *C. trachomatis* is possible and it is believed that it is due to a high Th1, IFN-*γ* response. The host cell and immune cells produce IFN-*γ* which decreases the amino acid tryptophan which is essential for chlamydia intracellular growth [39]. By pumping IFN-*γ* into the infected region, the immune effectors, *A*, help the cells recover from chlamydia infection, which we capture using the *γA* terms.

Infection dynamics were simulated in Mathematica [76] using NDSolve (with methods ‘BDF’ or ‘StiffnessSwitching’) for numerical integration.

### Parameter values and sensitivity analyses

Nearly all the parameter values could be set using data from the literature, which mostly lay in narrow ranges (Tables 1 and 3). Parameters for which we had little information were either kept free or calibrated. For instance we used Δ*q* to scale all equilibrium population sizes (see Results).

To test the robustness of our results, we performed uncertainty and sensitivity analyses using Latin Hypercube Sampling and Partial Rank Correlation Coefficients (PRCC) via the pse package in R [77], which is popular for disease models [78], and numerical integration was done using deSolve package. We generated 1,000 parameter sets by Latin Hypercube sampling from uniformly distributed parameter values within a specified biologically realistic range. PRCCs were calculated between the rank-transformed samples and the resulting output matrix of the response variables (e.g. duration of infection, maximum pathogen load). 100 bootstraps were performed to generate 95% confidence intervals. The magnitude of the PRCCs determines the effect strength of a given parameter on a specific response variable (0 for no effect and 1 for very strong) and the sign indicates whether the response grows or shrinks with increasing the parameter value.

Monotonicity for each parameter was checked for each response variable, and the parameter ranges were shortened when monotonicity was not obeyed. This was not common and was usually for values very close to zero.

### Parameter estimation from experimental data

We inferred parameter values from the data we collected over 3 weeks from a 3D raft culture of NIKS cells. Note that cells attached to the basal membrane were considered basal and those above them were counted as suprabasal cells. This was done (rather than use differentiation markers) in order to differentiate between true basal cells and parabasals and to estimate a carrying capacity, *N*_*b*_. Model parameters were inferred using maximum likelihood estimation and trajectory matching, assuming measurement error follows a Poisson distribution. Fitting and model predictions were performed in R software [79], using packages bbmle [80], deSolve [81], and pomp [82]. Note that the parameter values estimated experimentally were not used for the infection models since the experiments had the tissues growing up into full stratified form while infections usually start with the epithelium already at homeostasis, thus the epithelium parameters from the literature were more appropriate.

## Supporting information

S1 Fig Effect of re-seeding on wart-associated HPV infection kinetics. **a**. Infection with baseline parameters. **b**. Here infection rate of basal cells *β* decays to zero with time (with decay rate *b* = 0.05). No wart-like manifestation of the infection is possible.
(EPS)

S2 Fig Effect of parameter variations on the kinetics of HR-HPV infection. **a**. Infection with baseline parameters. **b. and c**. HR-HPV gives wart-like infections with either higher HPV-driven proliferation, (3x *α*_*b*_) or higher burst size (1 order of magnitude higher), thus demonstrating that HR-HPVs need to keep both of these parameters down in order to have flat, slow growing infections. **d**. Progression with stronger HPV-driven proliferation, increasing symmetric divisions biased toward making more basal-like cells, and increasing differentiated cell death.
(EPS)

S3 Fig Chlamydia dynamics. **a**. Time series of uninfected cells (*U*_*d*_ and *U*_*p*_) and the infected cells of the same layers (*I*_*d*_ and *I*_*p*_). Infection with baseline parameters.
(EPS)

S4 Fig Non-stratified HPV infection model schematic. A population of target cells, *T*, becomes infected by interacting with free virions, *V* at a rate *β*. Infected cells, *I*, self-proliferate, *ρ*_*a*_, due to HPV infection. Infected cells die naturally, *μ*, and release the virions they contain with a burst size of *θ*. Free virions are cleared by mucus, *ζ*, and infected cells are killed by the adaptive response, *A*, at rate *σ*.
(EPS)

S5 Fig Non-stratified HPV model. **A. Time series** of i. low cell proliferation driven by HPV infection (wart-associated-HPV-like), *ρ*_*a*_ = 0.7 and *θ* = 10^6^, and ii. high cell proliferation by HPV infection (HR-HPV-like), *ρ*_*a*_ = 1.4 and *θ* = 10^4^. Cell accumulation and duration are the opposite of what is seen in real infections, i.e. HR-HPV infections should accumulate less cells and last longer. **B, C, D. Parameter plots** of burt size, *θ*, and HPV-driven cell proliferation, *ρ*_*a*_. The magnitude of the peak of infected cells, *I*, (C) and the duration of the infection (D) are controlled almost exclusively by HPV-driven cell proliferation, *ρ*_*a*_, not burst size. Parameter values: *β* = 10^−10^, *N*_*b*_ = 10^3^, *μ* = 0.67, *ζ* = 1.18, *κ* = 0.0024, *σ* = 0.0001.
(EPS)

S1 Text Supporting information. Supplementary methods and results.
(PDF)

S1 Code Supporting code. R file that uses 3 csv data files for model fits.
(R) Click here for additional data file.

S2 Code Supporting code. Mathematica file that generates figures 3, 4, and supplementary figures.
(NB) Click here for additional data file.

S3 Code Supporting code. Mathematica file that generates figures for non-stratified model.
(NB) Click here for additional data file.

S1 Data Supporting data. CSV file.
(CSV)

S2 Data Supporting data. CSV file.
(CSV)

S3 Data Supporting data. CSV file.
(CSV)
