# Supplementary material for: Epithelial stratification shapes infection dynamics
Source: PLoS Comput Biol. 2019 Jan 23;15(1):e1006646. doi: 10.1371/journal.pcbi.1006646 (PMC6361466; doi:10.1371/journal.pcbi.1006646)
Supplement: S1 Text — Supplementary methods and results. (PDF) [file pcbi.1006646.s006.pdf]

Supporting Information for ‘Epithelial stratification affects infection dynamics’

Murall et al.

1. Supplementary Results

A. Analysis and parameter exploration.

A.1. Uninfected epithelium. The uninfected epithelium model is a linear system of ODEs that can be studied analytically. We use a continuous time model because the stages overlap in time and have different birth/death rates. The system has only one equilibrium, which is

$$\tilde{U}_s = \frac{(1-\Delta p)(1-\Delta q)}{-\Delta q}\,\frac{\rho_b N_b}{\mu} \quad [1a]$$

$$\tilde{U}_d = \frac{(1-\Delta p)(1-\Delta q)}{-\Delta q}\,\frac{\rho_b N_b}{\nu} \quad [1b]$$

$$\tilde{U}_p = \frac{1-\Delta p}{-\Delta q}\,\frac{\rho_b N_b}{\rho_p} \quad [1c]$$

Its Jacobian matrix is a diagonal matrix,

$$J = \begin{bmatrix} -\mu & \nu & 0 \\ 0 & -\nu & (1-\Delta q)\rho_p \\ 0 & 0 & \Delta q\ \rho_p \end{bmatrix} \quad [2]$$

and thus the eigenvalues are easily found ($\lambda_1 = -\mu$, $\lambda_2 = -\nu$, $\lambda_3 = \Delta q\ \rho_p$).

Since μ, ν, and ρ_p are biological parameters and must be positive, this equilibrium is stable iff $\Delta q\ \rho_p < 0$. For this condition to hold (and the epithelium size to make biological sense), Δq must be negative. The fact that the eigenvalues are never complex indicate that there are no limit cycles and that the solution grows (or decays) unbounded when the equilibrium is unstable.

The eigenvalues also allow us to calculate the relaxation time, which indicates which parameters have the most effect on how quickly the system reaches equilibrium (i.e. how quickly the epithelium reaches homeostasis). If the eigenvalues are all negative, the leading eigenvalue, λ_l, determines the long-term behaviour of the system since it has the largest relaxation time, $-1/\lambda_l$. Which eigenvalue is the leading eigenvalue changes depending on which parameter values are used. Therefore, we used a sensitivity analysis to study within our given parameter space which parameter has the most effect on time to equilibrium. Our results show that Δq is the most important parameter (Table 2 in main text).

Next we investigate what governs the thickness, or in other words the sum of the equilibria. Let h be a measure for how thick the epithelium is at homeostasis ($h = \tilde{U}_p + \tilde{U}_d + \tilde{U}_s$). To determine which parameters affect thickness the most, we maximize h. To do so, we simplify the model by making two assumptions: i) we do not follow U_s because it simply collects cells leaving the U_d population and ii) we re-parameterize the model, such that, $C_1 = \rho_b(1-\Delta p)N_b$, $C_2 = \rho_p\ \Delta q$, and $C_3 = \rho_p(1-\Delta q)$. Together this give the following simplified model,

$$\frac{\mathrm{d}U_d}{\mathrm{d}t} = C_3 U_p - \nu U_d \quad [3a]$$

$$\frac{\mathrm{d}U_p}{\mathrm{d}t} = C_1 - C_2 U_p. \quad [3b]$$

Using this set of equations, we get $h = \frac{C_3\ C_1}{C_2 \nu} + \frac{C_1}{C_2}$. By computing the partial derivative of h with respect to the 4 parameters and setting them to 0 (i.e. solving for $\frac{\partial h}{\partial C_2} = \frac{\partial h}{\partial C_3} = \frac{\partial h}{\partial C_1} = \frac{\partial h}{\partial \nu} = 0$), and then checking the sign of the second order derivatives for concavity, we find that there is no finite set of parameter values that maximizes h. Therefore, to study how much parameters affect the maximum of h in a restricted parameter space, we use uncertainty and sensitivity analysis (as described in the methods).

A.2. Parameter inference. In order to fix the carrying capacity of the basal layer, N_b, we choose the maximum value of the DAPI counts of the basal cells in FOVs across all replicates. Experiments that reach homeostasis within the experimental time window will best estimate this parameter. Given that Δp was estimated to be ≈ 0, this implies that a growing equation for the basal layer (equation 2 in the main text) is not necessary and future fits could just use the original model that assume a constant population size of U_b.

In order to reduce the number of parameters estimated, we used the counts of cells dividing at each time point from the BrDU stains to determine a relationship between the replication rates as a means. This constrained the value of ρ_p to be smaller than the replication rate of basal cells, ρ_b. The BrdU stains give the proportion of cells dividing in each layer (basal, non-keratinized suprabasal and keratinized suprabasal) at each measured time point and we assumed that this is represents the lit-up cells during the cell cycle stages of DNA systhesis (S), postsynthesis (G2) and mitosis (M). Following (1) the total time of the mean cell cycle, T_c, is the sum of the time in these three phases, T_r, and the time in postmitotic phase (G1), i.e. $T_c = T_r + T_{g1}$. The proportion of cells that are labeled and lit up (which we have from the BrdU data) are L_i, where $i = b$ or p for basal cells or parabasal cells respectively. We assume then that $L_i = T_r/T_{ci}$ and that T_r is constant (only the G1 phase can vary in duration) and therefore is the same for both basal and parabasal cells. Assuming the replication rates follow these relationships with the mean cell cycle time, $\rho_b = ln(2)/T_c$ and $\rho_p = ln(2)/T_c$, and substituting $T_{cb} = T_r/L_b$ and $T_{cp} = T_r/L_p$ we obtain the following relationship $\rho_p = (\frac{L_p}{L_b})\ \rho_b$. Given that the data does not distinguish parabasals from differentiated cells in the non-keratinized suprabasal layer, we correct this equation with the fraction of parabasal cells, $\frac{U_p}{U_p + U_d}$, so that ρ_p is not applied to differentiated cells. This gives the equation we used for parameter estimation,

$$\rho_p = \rho_b \left(\frac{L_p}{L_b}\right)\left(\frac{U_p}{U_p + U_d}\right), \quad [4]$$

where U_p and U_d are the variables parabasal and differentiated cells in Model 1 in the main text. The mean proportions of dividing cells for each cell type was calculated from the BrdU stains, giving $\frac{L_p}{L_b} = \frac{0.02}{0.15}$. Thus the basal layer had more dividing cells at any given time than parabasal layer. This relationship had a strong effect on the inferred Δq value and thus it should be estimated from the data for specific systems in future studies.

The inferred death rate, μ, was found to be nearly zero (Table 1, main text) which is consistent with the fact that in the experiment there is no mucus to wash away dead cells thus this parameter would expected to be zero experimentally (the dead cells pile up and would be counted).

Finally, using a loglikelihood ratio test, we found that for this dataset, a reduced model where $\Delta p = 0$ outperformed our full model, however, other reduced models where $\Delta q = 0$ did not, signifying then that Δp is not necessary but Δq is to explain our data.

A.3. Human papillomaviruses. For low-risk HPV infections, we show that re-seeding (via some infection of the basal cells during at least the initial part of the infection) is necessary for creating a wart-like excess number of cells. We make the infection rate a decaying function that approaches zero, i.e. $\beta(t) = \beta_0 e^{-bt}$, thus mimicking rapid microabrasion repair. In Figure S1b, we see that this quick removal of re-seeding decreases the number of infected cells (in this case, nearly 2 orders of magnitude lower than the baseline). As a consequence, the infection goes nearly undetected by the immune system and lasts for many years. Thus, if microabrasions repair quickly then higher burst sizes (i.e. HPV types with a colonization strategy) would be more adaptive.

In Figure S2a, b and c, we demonstrate how increasing either burst size or HPV-driven proliferation of basal/parabasal cells leads to faster growing infections and thus detection and clearance by the immune system. To model progression Figure S2, we made Δq, Δp, m and μ increase logistically with respect to time using the theta-logistic equation (2). Let N be a dummy variable, which can be replaced by Δq, Δp, m or μ, has the form

$$\frac{\mathrm{d}N}{\mathrm{d}t} = rN\left(1 - \left(\frac{N}{K}\right)^{\theta}\right) \quad [5a]$$

where $K = 1$ and $r = 0.01$. In order for the curves reach saturation more slowly $\theta = 1/2$. These new time variant equations were rescaled so that they increased within the following ranges: Δq [-0.012,0], Δp [-0.012,1], m [0.1,1] and μ [0.67,4]. In addition ρ_p was increased to 3. Note that this causes the parabasal cells to bulk-up. In any case, a full investigation into modelling progression of HR-HPV is beyond the scope of this paper. In particular it would require changing several assumptions over time and including a more dynamic interaction with the immune system.

A.4. Chlamydia. In Figure S3 we see the effect of when infection rates are different between the stages (i.e., $\beta_b < \beta_p < \beta_u$), namely that there is asynchrony of the oscillations between the uninfected cells and the infected cells. Comparing the dynamics of U_d and I_d or U_p and I_p helps visualize the time-lag between the oscillations. From a dynamical perspective, this echoes ecological predator-prey results (3): if a 'predator' (here chlamydia) feeds upon a stage-structured 'prey' population (here epithelium) with differing attack rates between the stages (here $\beta_b < \beta_p < \beta_u$), dampened oscillatory population dynamics can be observed. The time-lags between the epithelial layers and the asynchrony between the uninfectecd and infected epithelial layers stabilize the infection dynamics. If the infection rates are similar for all stages ($\beta_b = \beta_p = \beta_u$), the dynamics are less stable, with large oscillations around zero that generate infections with only acute phases.

A.5. Non-stratified HPV infection model. In order to compare what added benefit the stratification of the epithelium can give when it is explicitly considered in our model, we build a non-stratified HPV infection model. As in 'conical' viral dynamics models (4), we consider four main populations: target cells (which in this case are epithelium cells lumped into one population), T, infected cells, I, free virus particles, V, and a population of effector cells from the adaptive immune response, A. Assuming that the epithelium is at homeostasis when the infection begins, we assume T is at a carrying capacity and thus constant. We considered two other formulations where we had an explicit T equation: $\frac{dT}{dt} = \lambda\ T - \mu\ T$ or $\frac{dT}{dt} = \lambda\ T(1 - T/N_b)$, where N_b is a carrying capacity and $\lambda = \mu$. The results we present below did not change significantly when using either of these two T equations (not shown), thus we present here the simplest version of the model. The three equations of the system are

$$\frac{\mathrm{d}I}{\mathrm{d}t} = \beta\ V\ T + \rho_a\ I - (\mu + \kappa\ A)\ I \quad [6a]$$

$$\frac{\mathrm{d}V}{\mathrm{d}t} = \mu\ \theta\ I - \zeta\ V \quad [6b]$$

$$\frac{\mathrm{d}A}{\mathrm{d}t} = \sigma\ I\ A. \quad [6c]$$

The epithelium cells are infected by free virions at a rate $\beta\ V\ T$. HPV drives infected cells to proliferate, $\rho_a\ I$. Infected cells die at a natural rate of $\mu\ I$ and are killed by the interaction of immune cells, $\kappa\ A\ I$. Free virions are released when infected cells die naturally, $\mu\ \theta\ I$ and are cleared by the mucus at rate $\zeta\ V$. Finally, immune cells proliferate as a response to the density of infected cells, $\sigma\ I\ A$. The model schematic is below (Figure S4). We maintained the parameter estimates the same as the ones used in the main text (Table 3), except we varied ρ_a and θ to study their effect on the dynamics of the infection. The main assumption contrasting wart-associated and HR types is that HR ρ_a is higher (due to their stronger cell transforming properties).

We find that the non-stratified model of HPV infection is not able to reproduce what is known about HPV infections, particularly when comparing wart-associated vs HR infections. HR-HPV types have stronger cell proliferation properties than wart-associated types, however, they produce flat infections that, on average, last longer. The non-stratified model gives a contradictory result: the HPV-proliferation rate, ρ_a has a strong effect on infection dynamics such that the stronger it is the more infected cells accumulate and quickly (Figure S5 A and B-D). Therefore, the model erroneously predicts that HR-HPVs accumulate more cells and have shorter infections (Figure S5 A i vs. ii).

1. Averette HE, Weinstein GD, Frost P (1970) Autoradiographic analysis of cell proliferation kinetics in human genital tissues. i. normal cervix and vagina. *Am J Obstet Gynecol* 108(1):8–17.
2. Gilpin M, Ayala F (1973) Global Models of Growth and Competition. *Proceedings of the National Academy of Sciences* 70(12):3590–3593.

3. Murdoch WW, Briggs CJ, Nisbet RM (2003) *Consumer-Resource Dynamics*. (Princeton University Press, New Jersey), p. 462.
4. Perelson AS (2002) Modelling viral and immune system dynamics. *Nat. Rev. Immunol.* 2(1):28–36.

SI Figures

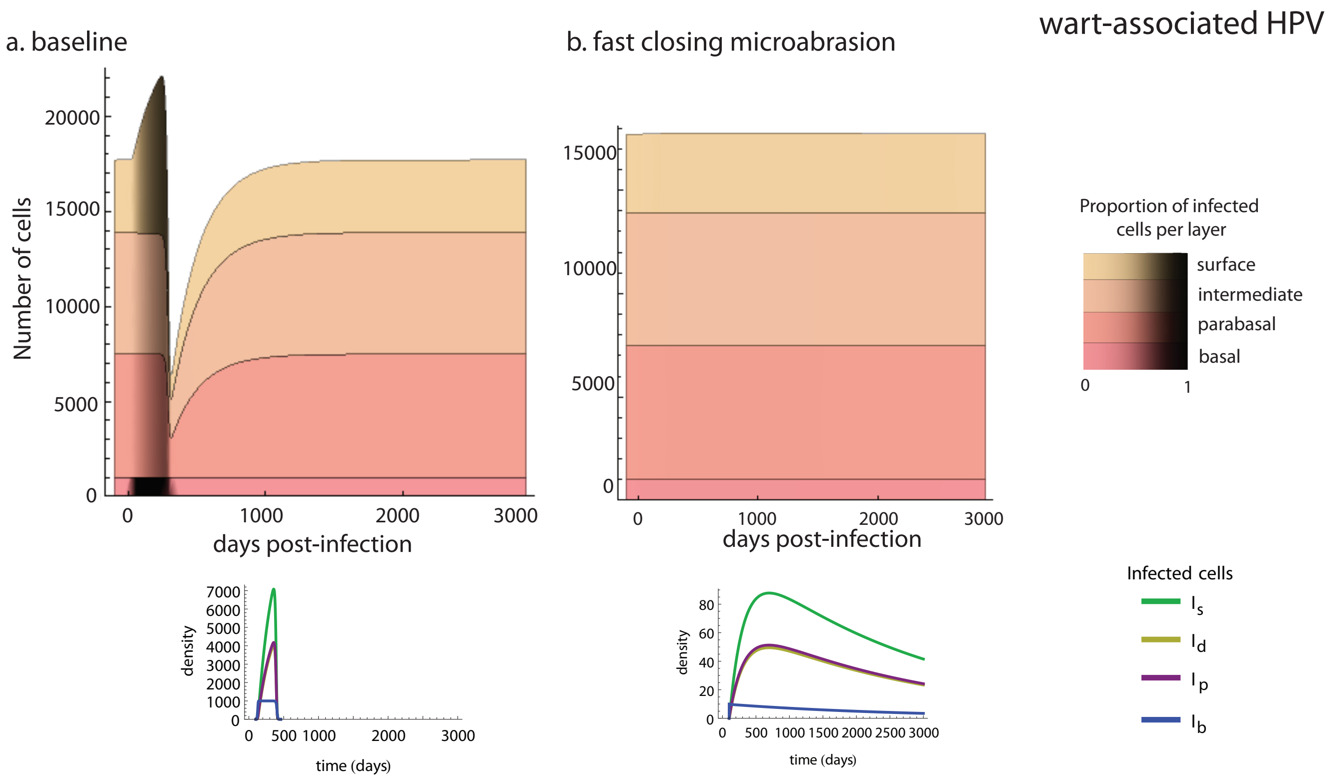

Fig. S1. Effect of re-seeding on wart-associated-HPV infection kinetics. **a.** Infection with baseline parameters. **b.** Here infection rate of basal cells β decays to zero with time (with decay rate $b = 0.05$). No wart-like manifestation of the infection is possible.

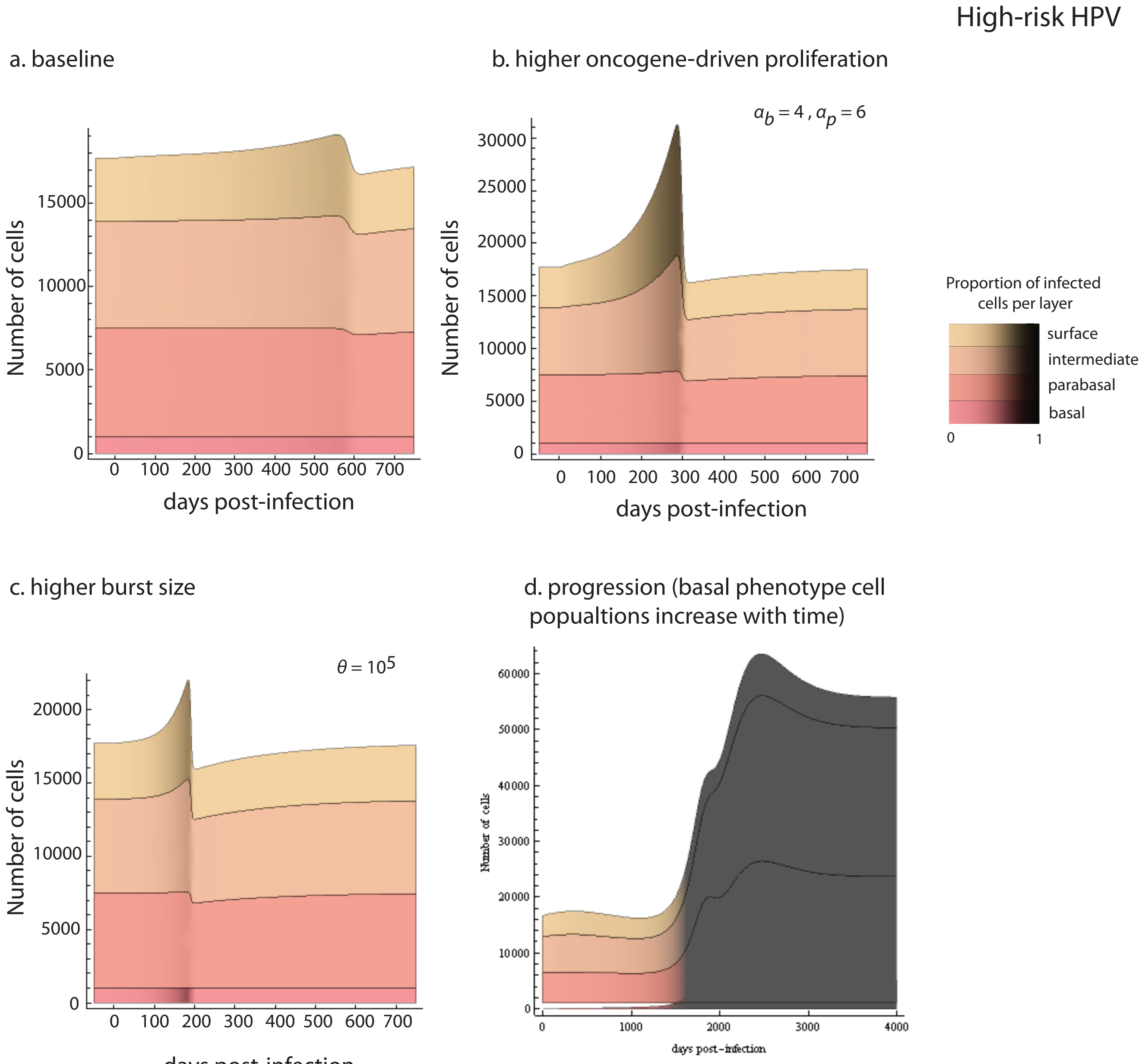

Fig. S2. Effect of parameter variations on the kinetics of HR-HPV infection. **a.** Infection with baseline parameters. **b. and c.** HR-HPV gives wart-like infections with either higher HPV-driven proliferation (3x) or higher burst size (1 order of magnitude higher), thus demonstrating that HR-HPVs need to keep both of these parameters down in order to have flat, slow growing infections. **d.** Progression with stronger HPV-driven proliferation, increasing symmetric divisions biased toward making more basal-like cells, and increasing differentiated cell death.

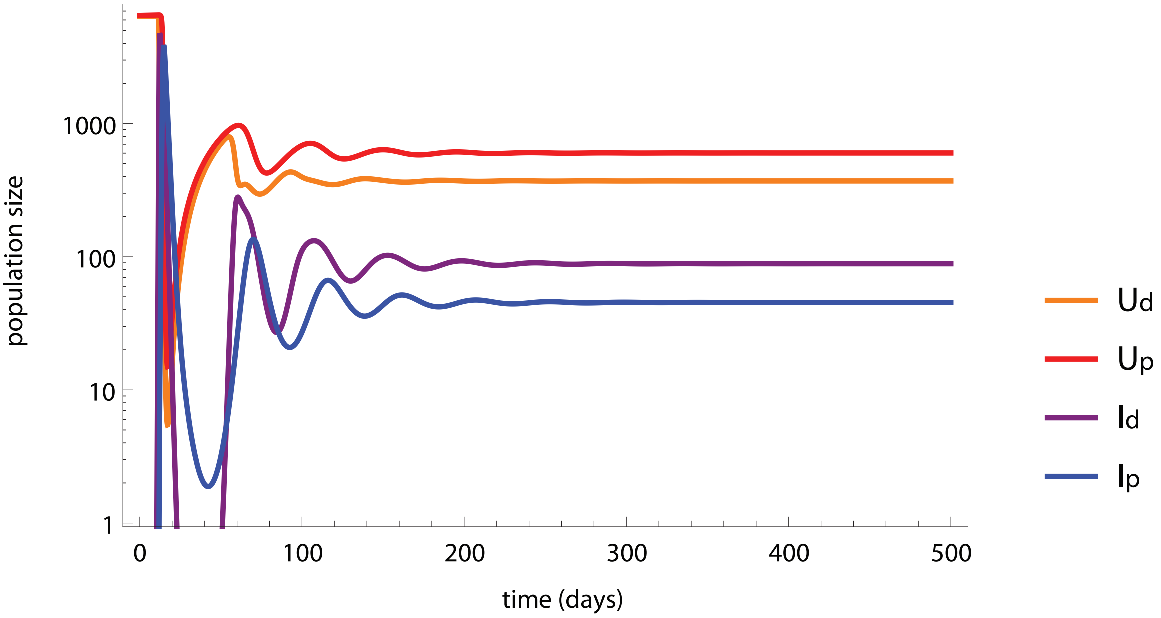

Fig. S3. Time series of uninfected cells (U_d and U_p) and the infected cells of the same layers (I_d and I_p). Infection with baseline parameters.

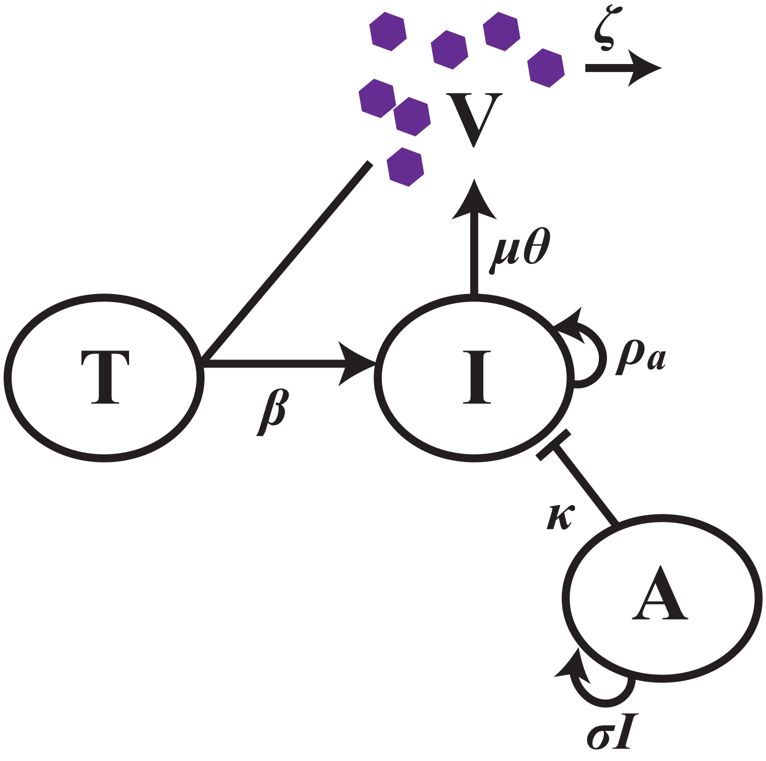

Fig. S4. Non-stratified HPV infection model schematic. A population of target cells, T, becomes infected by interacting with free virions, V at a rate β. Infected cells, I, self-proliferate, ρ_a, due to HPV infection. Infected cells die naturally, μ, and release the virions they contain with a burst size of θ. Free virions are cleared by mucus, ζ, and infected cells are killed by the adaptive response, A, at rate σ.

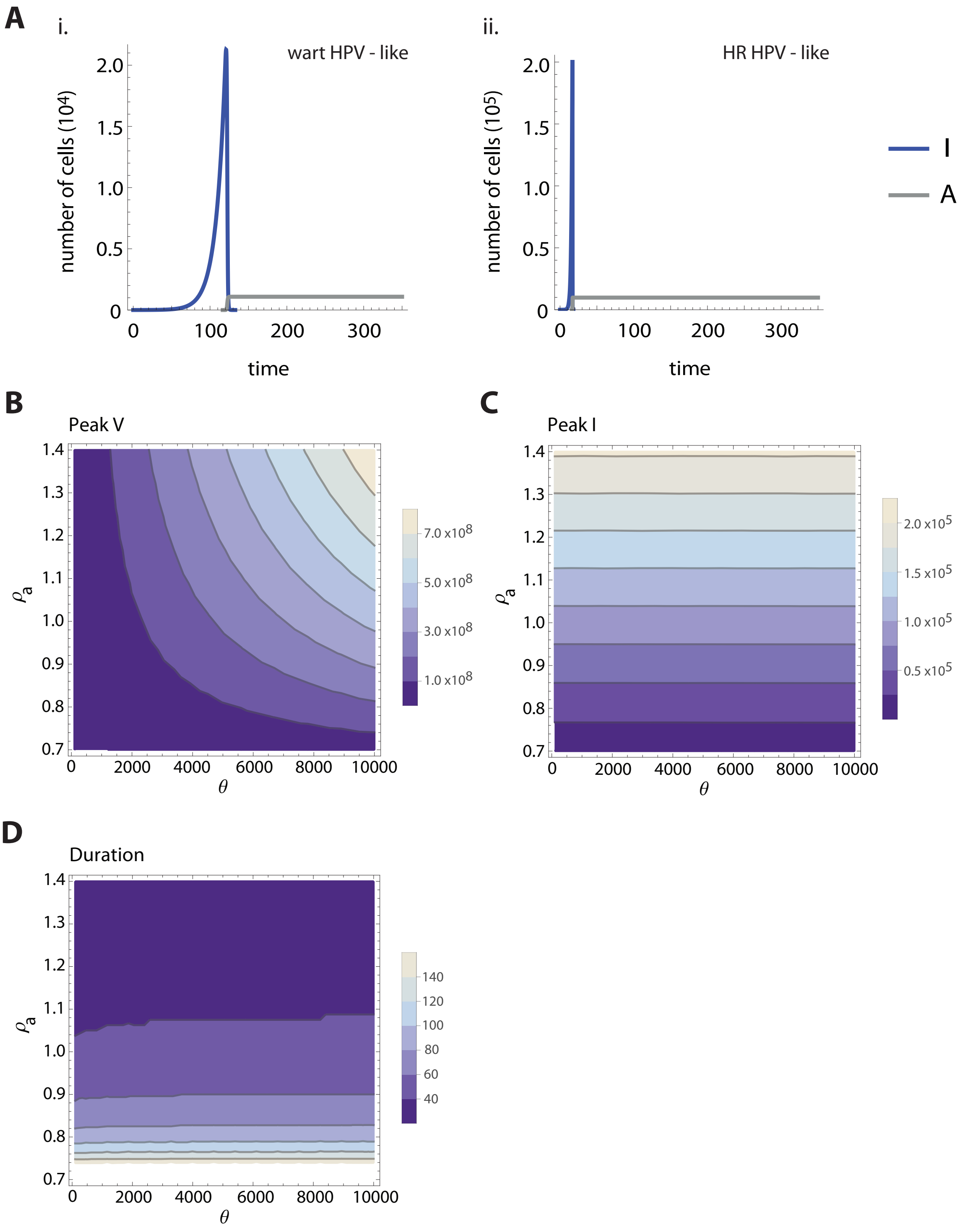

Fig. S5. Non-stratified model. A. Time series of i. low cell proliferation driven by HPV infection (wart-associated-HPV-like), $\rho_a = 0.7$ and $\theta = 10^6$, and ii. high cell proliferation by HPV infection (HR-HPV-like), $\rho_a = 1.4$ and $\theta = 10^4$. Cell accumulation and duration are the opposite of what is seen in real infections, i.e. HR-HPV infections should accumulate less cells and last longer. **B, C, D. Parameter plots** of burt size, θ, and HPV-driven cell proliferation, ρ_a. The magnitude of the peak of infected cells, I, (C) and the duration of the infection (D) are controlled almost exclusively by HPV-driven cell proliferation, ρ_a, not burst size. Parameter values: $\beta = 10^{-10}$, $N_b = 10^3$, $\mu = 0.67$, $\zeta = 1.18$, $\kappa = 0.0024$, $\sigma = 0.0001$
